# Personal Hygiene Practices among Urban Homeless Persons in Boston, MA

**DOI:** 10.3390/ijerph14080928

**Published:** 2017-08-18

**Authors:** Jessica H. Leibler, Daniel D. Nguyen, Casey León, Jessie M. Gaeta, Debora Perez

**Affiliations:** 1 Department of Environmental Health, Boston University School of Public Health, 715 Albany St, T430W, Boston, MA 02118, USA; ddn@bu.edu (D.D.N.); dperez@bu.edu (D.P.); 2 Boston Health Care for the Homeless Program, 780 Albany St. Boston, MA 02118, USA; cleon@bhchp.org (C.L.); jgaeta@bhchp.org (J.M.G.); 3 Department of Medicine, Boston University School of Medicine, 72 E. Concord St. Boston, MA 02118, USA

**Keywords:** homeless, hygiene, sanitation, street people, hand hygiene, self-care

## Abstract

Persons experiencing homelessness in the United States experience significant barriers to self-care and personal hygiene, including limited access to clean showers, laundry and hand washing facilities. While the obstacles to personal hygiene associated with homelessness may increase risk of infectious disease, hygiene-related behaviors among people experiencing homelessness has received limited attention. We conducted a cross-sectional study of individuals experiencing homelessness in Boston, MA (*n* = 194) to identify hygiene-related self-care practices and risk factors for reduced hygiene in this population. Most participants (72%) reported taking a daily shower. More than 60% reported hand washing with soap five or more times each day, and use of hand sanitizer was widespread (89% reported using sanitizer in the last week). A majority (86%) used a laundromat or laundry machine to wash clothing, while 14% reported washing clothing in the sink. Heavy drinking, injection drug use, and sleeping outdoors were identified as significant risk factors for reduced hygiene practices. People experiencing homelessness who also engage in these activities may be among the most difficult to reach for intervention, yet targeted efforts may decrease illness risk associated with reduced hygiene. Housed friends and family play a critical role in assisting homeless individuals maintain hygiene by providing showers and laundry facilities.

## 1. Introduction

Access to adequate sanitation infrastructure, including toilets, showers and hand washing facilities, has long been identified as a precursor for personal hygiene and good health [1,2]. Persons experiencing extreme poverty, especially those experiencing homelessness, may face reduced access to sanitation facilities and difficulties engaging in health-promoting self-care activities as a result. Reduced availability of public toilets in the United States in the last decade has a disproportionately negative impact on hygiene among the very poor persons who utilize these facilities for daily self-care [3,4]. While good personal hygiene is well known to reduce risk of infectious disease and improve mental health, access to sanitation facilities and hygiene behaviors among people experiencing homelessness in the United States have received little attention [5,6,7,8,9]. As prevalence of homelessness increases in the United States, the availability of adequate sanitation facilities, and the utilization of these facilities, become increasingly relevant for public health [10].

A substantial body of literature documents the association between sanitation infrastructure and infectious diseases, specifically diarrheal diseases such as cholera and typhoid [11,12]. In high-income countries, despite substantial investment in sanitation infrastructure and the near-elimination of many waterborne diseases, very poor persons and individuals experiencing homelessness may lack regular access to bathrooms and showering facilities. In addition to limited access, individuals may feel uncomfortable or unsafe using public facilities, such as showers in emergency shelters, which may reduce utilization of these facilities, despite their availability [13,14]. Resource constraints, especially among public emergency shelters and day centers, may result in low levels of cleanliness or reduced disinfection in public bathroom facilities [15]. Additionally, persons experiencing homelessness experience higher prevalence of mental illness and substance use than housed persons, which pose overall challenges in meeting basic needs of daily living, including an ability to maintain personal hygiene [16,17,18,19,20]. In these ways, challenges associated with self-care are a hallmark of the homeless experience [6,21].

Reduced hygiene-related self-care practices among people experiencing homelessness in high-income countries have been associated with a variety of communicable and non-communicable disease outcomes. Infrequent showers and laundering clothing has been associated with increased risk of ectoparasite infestation (i.e., body lice, fleas, head lice, and scabies) among people experiencing homelessness in a large study in Europe [22]. Sleeping outdoors has been identified as a particular risk factor for increased prevalence of scabies and body lice [23]. In addition to causing discomfort, some ectoparasites, notably body lice, transmit vector-borne infectious agents, including *Bartonella quintana*, *Rickettsia akari* and *Yersinia pestis*, which have been identified among some populations of people experiencing homelessness in urban areas and may pose long-term health consequences for some individuals [24,25]. The severity of these infections may be intensified by co-morbid conditions and reduced access to early treatment among people experiencing homelessness [26].

Reduced hygiene is also a risk factor for skin infections, notably methicillin-resistant *Staphylococcus aureus* (MRSA), among urban poor and marginalized persons, notably injection drug users [27,28,29]. MRSA is transmitted in crowded environments through close personal contact, and people experiencing homelessness experience elevated incidence of MRSA presumably due to contact transmission within sanitation facilities and crowded living conditions [30,31,32]. These infections may have particular severity for persons with limited access to follow-up care, and pose burdens specifically on emergency medicine departments where many people experiencing homelessness receive medical care [33,34].

Limited access to sanitation may also exacerbate chronic diseases, such as HIV or diabetes, by posing barriers to treatment adherence [35,36,37]. Patients who return to emergency shelters following medical interventions, such as surgery, may face increased risk of post-operative infection or other complication due to limited access to clean facilities. People experiencing homelessness may be more likely to experience recurrent skin infection following treatment as well, possibly due to challenges in maintaining skin hygiene, reinfection from continued exposure to contaminated surfaces in the living environment, or lack of follow-up care [38].

Personal hygiene has also been identified as a positive contributor to mental health among persons experiencing homelessness. In Rosengard et al., 66% of homeless individuals reported bathing every day and respondents identified “taking a shower after several days without one” as being extremely important to them [39]. In a study of risk factors for negative physical and mental health in homeless men with HIV, reduced access to bathrooms was identified as a significant risk factor for poor physical and mental health [40]. Elevated odds of poor self-rated health was found among men who reported not showering regularly [13]. Personal hygiene and self-care, particularly access to regular showering and clean clothing, may also increase likelihood of transitioning out of homelessness, through obtaining a job or a housing placement [41,42,43,44].

Despite the importance of personal hygiene and the challenges faced by people experiencing homelessness in maintaining personal hygiene, few studies have explored hygiene-related self-care practice among persons experiencing homelessness in high-income countries. We conducted a cross-sectional study in Boston, MA to identify hygiene practices and use of sanitation facilities among people experiencing homelessness in this large city in the United States. The goal of this study was to inform future efforts to improve access to sanitation facilities and target interventions to improve hygiene-related health in this hard-to-reach and vulnerable population.

## 2. Materials and Methods

Study design: A cross-sectional study of individuals experiencing homelessness in Boston, MA was conducted in July 2015. Participants were recruited in the patient waiting area and clinic lobby of a large urban healthcare provider for homeless persons, the Boston Health Care for the Homeless Program (BHCHP), during four daylong enrollment events. Inclusion criteria were current patient status at BHCHP, older than 18 years of age, and English-speaking. Private interviews were conducted with each participant using a validated questionnaire tool, and participation was anonymous. The questionnaire included a basic medical history, questions regarding recent shelter, duration of homelessness, hygiene, substance use and demographics. This study was nested within a larger study of skin infection risk among people experiencing homelessness. All aspects of study design and conduct were approved by the Institutional Review Board of Boston University Medical Campus (H-33761).

Participants were asked about daily frequency of hand washing with soap (never, once, 2–4 times, or ≥5 times), frequency of showering in the last week (never, once/week, twice per week, 3–6 times/week, or daily) and frequency of laundering clothing in the last month (never, 1–2 times, 3–5 times, or ≥6 times). Subjects were asked how they laundered their clothing (sink without soap, sink with soap, laundromat, shelter machine/service, friend’s house or other), and whether they shared clothing, towels or bedding with other people. Participation took approximately 20 min, and participants received a $10 gift card to a local pharmacy following study enrollment.

Statistical analysis: Questionnaire data were coded and inputted into Microsoft Access, then imported into Stata/SE 13.1 for analysis. Descriptive analyses regarding four hygiene behaviors (frequency of showering, hand hygiene, frequency and method of clothes laundering, and sharing clothes or bedding) were conducted. Showering data and hand washing data were transformed into binary variables to represent daily showering (Y/N) and frequent hand washing (≥5 washes/day, Y/N). Data on laundry (ever or never washing clothing in the last month, Y/N; using a washing machine, Y/N) and sharing clothing or bedding (Y/N) were also analyzed as binary.

Univariate logistic regression models were used to evaluate the association between the hygiene behaviors and behavioral covariates, including heavy drinking (defined as drinking ≥5 days per month to intoxication, per the Substance Abuse and Mental Health Services Administration), injection drug use in the last year, sleeping outdoors most frequently during the last week, and sleeping in emergency shelters most frequently during the last week [45].

To account for small cell counts, an index variable representing reduced hygiene was developed to use as an outcome for multivariable log linear (Poisson) regression. One point was assigned to each of the following responses and aggregated for the index variable: not showering daily, infrequent daily hand washing or sanitizer use (<5 times), never washing clothing during the last month, washing clothing in the sink with or without soap (instead of a washing machine), and sharing clothing or bedding. Stepwise backwards selection was used to identify significant predictors in the multivariable regression, including demographic covariates of age and gender.

## 3. Results

During enrollment, 194 persons met inclusion criteria and were enrolled. Eleven persons were recruited but not enrolled for not meeting inclusion criteria. The average age of participants was 48 years old, and 54% were female (*n* = 105) (Table 1). Sample demographics reflected those of the clinical population overall, with approximately 41.8% of study participants African American/black and 18.7% Hispanic/Latino, compared to 37.9% and 19.1% across the clinical population [46]. During the last month, 54% percent (*n* = 104) reported staying most frequently in an emergency shelter, 7% (*n* = 14) slept on the street, 14% (*n* = 27) stayed with friends or family, and 20% (*n* = 39) slept most frequently in supportive housing or in housing with no support (Table 1). Fifteen percent of participants (*n* = 30) reported drinking alcohol heavily and 28% (*n* = 54) said they had injected drugs in the past year.

Seventy-two percent (*n* = 139) of subjects reported showering or bathing daily (Table 1). Twenty-one percent reported showering 3–6 times each week (*n* = 40), and 7.2% said they showered 1–2 time per week (*n* = 14). One individual reported not showering or bathing at all in the last week. The majority of participants showered at an emergency shelter (59%; *n* = 112). Twenty percent (*n* = 38) reported showering at homes of friends or family and another 20% said they showered at other service facilities for people experiencing homelessness, such as day shelters.

Sixty-one percent of participants (*n* = 118) reported hand washing five or more times a day (Table 1). Approximately 37% said they washed their hands 2–4 times per day (*n* = 71), and five participants (2.6%) reported washing their hands once or not at all during the day. Eight nine percent (*n* = 173) reported use of hand sanitizer in the last week.

The majority of participants washed clothes 1–2 times per month (63%; *n* = 123). Five percent (*n* = 9) reported not washing clothes at all in the last month. The majority of participants (86%, *n* = 167) reported using a washing machine and dryer (including a laundromat, shelter laundry facility or machines at the house of friends/family) for their personal laundry. Fourteen percent (*n* = 27) of participants said they washed their laundry in the sink, of which 10 of these individuals (5% of total) said they laundered their clothing in the sink without soap. Nearly 30% of participants (28%; *n* = 55) reported sharing clothes or bedding. Items shared included blankets, pajamas, t-shirts, and sheets.

In univariate analyses, significant predictors for not showering daily were heavy drinking (OR: 5.15, 95% CI: 2.3, 11.7, *p* < 0.0001) and sleeping outdoors (OR: 3.2, 95% CI: 1.1, 9.3, *p* = 0.03) (Table 2). Injecting drugs in the past year was significantly associated with infrequent hand washing (OR: 2.8, 95% CI: 1.5, 5.5, *p* = 0.001), sharing clothing/bedding (OR: 2.5 (1.3, 4.9, *p* = 0.007) and laundering clothing using only the sink instead of a washing machine (OR: 2.4; 95% CI: 1.0, 5.5; *p* = 0.04). Outdoor sleeping was also positively associated with sharing clothing or bedding (OR: 3.2; 95% CI: 1.1, 9.3; *p* = 0.03). No significant associations between other behavioral or demographic variables and the hygiene outcomes were observed in univariate analyses.

In the multivariable log linear model, heavy drinking (*p* = 0.05), injection drug use in the last year (*p* = 0.01) and sleeping outdoors (*p* = 0.01) were significantly associated with reduced hygiene. Transience, sleeping in shelters, age and gender were not significantly associated with reduced hygiene in this model.

## 4. Discussion

Self-care behaviors, including good hygiene, are critically important for individuals navigating homelessness. A majority of persons experiencing homelessness in our study engage in regular bathing or showering, frequent hand washing and regular laundering of personal clothing and articles. Use of hand sanitizer is nearly universal in this population (89%). While our data likely reflect a subset of the population of people experiencing homelessness in Boston—those who seek out health care services—our findings reflect positively on availability and utilization of sanitation resources available in Boston.

Our analysis identified key risk factors for reduced hygiene—substance use and sleeping outdoors—which could be used to target interventions towards persons at risk of hygiene related health complications. Persons who engaged in heavy drinking were significantly less likely to take a daily shower compared to individuals who did not drink heavily. Injection drug use was associated with a number of reduced hygiene-related outcomes, including not showering daily, infrequent hand washing, sharing clothing or bedding and laundering clothing in the sink. Emergency shelters often have policies in place to deny entry to individuals who appear to be under the influence of drugs or alcohol, which may result in reduced access to hygiene facilities among people with substance dependence. Substance use has also been associated with reduced self-care behaviors among people experiencing homelessness in prior research [47]. The role of mental health as a confounder of the relationship between substance use and reduced self-care and hygiene is an important area for future research. Relatedly, the role of improving access to sanitation facilities and self-care utilization may affect willingness to engage in treatment for substance use disorders. The intersection of these issues warrants follow-up.

Individuals who sleep on the street also reported fewer hygiene-related self-care practices in our study. This finding reflects both the predictors of street sleeping, the strongest of which is mental illness, as well as the reduced access to facilities associated with homelessness services, such as shelters [48,49]. Our study sample reflected street sleepers recruited at a health clinic, who are perhaps more engaged in self-care than street sleepers who do not engage with health services. Improving hygiene among health care seeking persons who sleep on the street reflects the real challenges in improving self-care behaviors in this difficult-to-reach population [50]. For some individuals, shared living areas and sanitation facilities feel unsafe and uncomfortable, which in turn may drive these persons to sleep outdoors. The role of access and utilization of sanitation facilities as a risk factor for street sleeping is an important area for future study, especially because such factors may be modifiable in some circumstances.

Adequate laundry facilities are important in reducing prevalence and transmission of ectoparasites and bacterial infections. Clean clothing also may reduce experiences of stigma associated with homelessness and may aid in the transition out of homelessness, by increasing likelihood of job placement, for example. Expanded laundry services at shelters or vouchers for laundromat use could improve utilization. Similarly, expanded access to free, clean bedding and clothing may reduce sharing of these items. Such services could be advertised at locations where individuals with substance abuse may frequent, including needle exchanges or addiction clinics.

People experiencing homelessness in this study reported significant usage of homes of friends and family members for hygiene behaviors, specifically showers and laundry. Visiting or staying overnight periodically at the homes of friends and family members allows for breaks from the shelter system and may also allow additional opportunities to maintain self-care and hygiene. The availability of hospitable friends and family members may be critical to allow persons experiencing homelessness to maintain adequate levels of hygiene and self-care, and is an important area for further research. Notably, persons with substance use disorders and/or mental health disorders may experience difficulty maintaining these relationships, which may explain the relatively reduced hygiene practices among heavy drinkers and injection drug users in our study.

Sample size was a central limitation of our study, and this research was not powered to address all associations of interest satisfactorily. Participants were volunteers who were seeking care at a health clinic, so are likely more engaged in self-care than persons who did not actively seek care. Because of this selection bias, extending these findings to the general population of persons experiencing homelessness should be done with caution, and our findings may overestimate hygiene practices of all people experiencing homelessness in Boston. More nuanced questions regarding laundering in particular may have uncovered important detail, for example, whether people purchased or received new clothes instead of laundering their clothes. Individuals may have felt ashamed or stigma associated with reduced hygiene, which led to over-reporting in showering and hand hygiene data in particular, for example. Larger studies are necessary to distill these factors. While cost prohibitive for this study, a larger study that included a comparison group of persons not experiencing homelessness would provide valuable information on baseline hygiene among the general population, an area in which there is limited data to date.

Hygiene behaviors among people experiencing homelessness likely reflect the availability of sanitation facilities, perception as to the safety or usability of these facilities, as well as personal practices. Our standardized questionnaire tool, while useful for generating preliminary data, was not adequate to address the multiple, interacting factors that give rise to how people feel about their environments and how they engage in self-care. Given the complexity of drivers of self-care behaviors, a qualitative study would be best suited to disentangle the motivations for hygiene practices in this population, and is recommended. Despite these limitations, this study engaged in in-person interviews of an understudied and vulnerable population already engaged within the health care system. The demographics of study participants parallel those of the Boston Health Care for the Homeless clinic patient population more generally, indicating a random sample.

## 5. Conclusions

Our findings suggest that while a majority of persons expressed appropriate hygiene-related self-care activities, a focused minority—persons who sleep outdoors and those who engage in substance use—experience reduced hygiene and self-care challenges. These populations should be targeted with resources to improve self-care in particular. Future work on individuals’ experiences using publicly available sanitation services is imperative, and was not addressed in our research. The role of family members and friends in assisting people experiencing homelessness to maintain personal hygiene is also an important area for future study.

## Figures and Tables

**Table 1:** Select demographics of study participants (*n* = 194).

Characteristic	Prevalence % (*n*) *
**Gender and race/ethnicity**	
Female	56.0 (108)
Black	41.8 (81)
Hispanic	18.7 (36)
Most frequent location for sleep in the last week *	
Homeless shelter	53.6 (104)
Street/outdoors	7.2 (14)
Supportive or transitional housing	11.3 (22)
With a friend or family member	13.9 (27)
Housing without support services	8.8 (17)
**Self-reported behaviors**	
Injection drug use in last year	27.8 (54)
Heavy drinker ^+^	15.5 (30)
Housed in the past 30 days *	
Homeless shelter	53.6 (104)
Street/outdoors	7.2 (14)
Doubled up	13.9 (27)
Supportive Housing	8.8 (17)
Housing with no support	8.8 (17)
Transitional/Treatment program	2.6 (5)
**Frequency of showers (in last week)**	
Daily	71.7 (139)
Not daily	28.4 (55)
**Most common location of shower**	
Shelter	59.0 (112)
Friends/family	20 (38)
Other homeless service provider	20 (38)
**Hand washing (daily frequency)**	
≥5 times	60.8 (118)
<5 times	39.2 (76)
Hand sanitizer used in the last week (yes)	89.2 (173)
Clothes/bedding sharing (yes)	28.4 (55)
**Laundry frequency (per month)**	
Never	4.6 (9)
1–2 times	63.4 (123)
3–5 times	23.2 (45)
≥6 times	8.8 (17)
**Laundry method**	
Laundromat	41.8 (81)
Shelter washing machine	22.2 (43)
Sink with soap	8.8 (17)
Sink without soap	5.2 (10)
Washing machine at home of family or friend	22.2 (43)

* Percentages may not sum to 100% due to missing data or response of “other” (data not shown); ^+^ Defined per SAMSA as drinking to intoxication more than five days during the last 30 days.

**Table 2:** Select risk factors for reduced hygiene behaviors among homeless persons in Boston, MA.

Hygiene Outcomes	Odds Ratio ^#^ (95% CI); *p*-Value		
	Heavy Drinking	Sleeping Outdoors	Injection Drug Use
Not showering daily	5.2 (2.3, 11.7); *p* < 0.001 *	3.2 (1.1, 9.3); *p* = 0.03 *	1.8 (0.9, 3.5); *p* = 0.10
Infrequent hand washing	1.0 (0.5, 2.3); *p* = 0.90	2.5 (0.9, 7.4); *p* = 0.09	2.8 (1.5, 5.5); *p* = 0.001 *
Sharing clothing or bedding	1.3 (0.6, 3.0); *p* = 0.50	3.2 (1.1, 9.3); *p* = 0.03 *	2.5 (1.3, 4.9); *p* = 0.007 *
Laundering using only sink	2.2 (0.8, 5.8); *p* = 0.11	0.9 (0.2, 4.4); *p* = 0.90	2.4 (1.0, 5.5); *p* = 0.04 *
Low hygiene (aggregate index variable) ^^^	1.4 (1.0, 1.9); *p* = 0.05 *	1.7 (1.1, 2.5); *p* = 0.01 *	1.4 (1.1, 1.8); *p* = 0.01 *

^#^ Results from univariate logistic regression models, with the following outcomes: not showering daily, infrequent hand washing, sharing clothing or bedding and laundering using only the sink. Heavy drinking, sleeping outdoors and injection drug use were considered as risk factors. * Statistically significant at *p* ≤ 0.05; ^^^ Multivariable log linear models using a hygiene index as an outcome, controlling for age and gender.
